# Bait‐ER: A Bayesian method to detect targets of selection in Evolve‐and‐Resequence experiments

**DOI:** 10.1111/jeb.14134

**Published:** 2022-12-21

**Authors:** Carolina Barata, Rui Borges, Carolin Kosiol

**Affiliations:** ^1^ Centre for Biological Diversity University of St Andrews St Andrews UK; ^2^ Institute of Population Genetics Wien Austria

**Keywords:** Bayesian inference, E&R, Moran model, pool‐seq, selection coefficients, targets of selection

## Abstract

For over a decade, experimental evolution has been combined with high‐throughput sequencing techniques. In so‐called Evolve‐and‐Resequence (E&R) experiments, populations are kept in the laboratory under controlled experimental conditions where their genomes are sampled and allele frequencies monitored. However, identifying signatures of adaptation in E&R datasets is far from trivial, and it is still necessary to develop more efficient and statistically sound methods for detecting selection in genome‐wide data. Here, we present Bait‐ER – a fully Bayesian approach based on the Moran model of allele evolution to estimate selection coefficients from E&R experiments. The model has overlapping generations, a feature that describes several experimental designs found in the literature. We tested our method under several different demographic and experimental conditions to assess its accuracy and precision, and it performs well in most scenarios. Nevertheless, some care must be taken when analysing trajectories where drift largely dominates and starting frequencies are low. We compare our method with other available software and report that ours has generally high accuracy even for trajectories whose complexity goes beyond a classical sweep model. Furthermore, our approach avoids the computational burden of simulating an empirical null distribution, outperforming available software in terms of computational time and facilitating its use on genome‐wide data. We implemented and released our method in a new open‐source software package that can be accessed at https://doi.org/10.5281/zenodo.7351736.

## Graphical abstract

We present Bait‐ER ‐ a method for estimating selection in allele frequency trajectory data from E&R experiments. Bait‐ER models allele frequencies through time in a population with overlapping generations. Our method performs well in both simulated and real time series data and it tests for selection without the need for simulating an empirical null distribution.

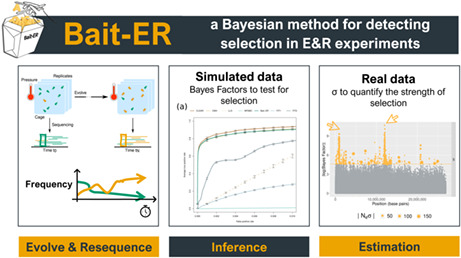

## 1 INTRODUCTION

Natural selection is a complex process that can dramatically alter phenotypes and genotypes over remarkably short timescales. Researchers have successfully tested theoretical predictions and collected evidence for how strong laboratory selection acting on phenotypes can be. However, it is not as straightforward to measure selection acting on the genome. Many confounding factors can lead to spurious results. This is particularly relevant if we are interested in studying how experimental populations adapt to laboratory conditions within tens of generations, in which case we need to take both experiment‐ and population‐related parameters into account.

**FIGURE 1:**
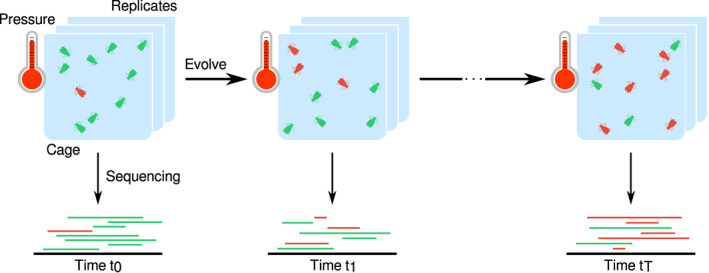
Example of an E&R experimental setup. E&R experiments expose several replicated populations (e.g., of flies, yeast, viruses) to a selective pressure (e.g., temperature, food regimes) for a specific number of generations *t*_*N*_. The replicated populations are surveyed at several time points by whole‐genome sequencing, which allows one to quantify changes in allele frequencies over time.

A powerful approach to gathering data on the genomics of adaptation is to combine experimental evolution, where populations are exposed to a controlled laboratory environment for some number of generations (Kawecki et al., 2012), with genome resequencing throughout the experiment. This approach is referred to as Evolve‐and‐Resequence (E&R, Figure 1). E&R studies are becoming increasingly more common and have already made remarkable discoveries on the genomic architecture of short‐term adaptation. Examples of experimental evolution studies include those on yeast (Burke et al., 2014), red flour beetles (Godwin et al., 2017) and fruit flies (Debelle et al., 2017; Turner et al., 2011). The E&R set‐up allows for describing the divergence between experimental treatments whilst accounting for variation amongst replicate populations (Schlötterer et al., 2015). This is true both at the phenotype and genotype levels. Consequently, the optimal approach to finding signatures of selection is to not only monitor allele frequency changes but to also search for consistent changes across replicates. Moreover, experimental populations are often sampled and pooled for genome sequencing. The motivation for sequencing pooled samples of individuals (pool‐seq) is that it is cost‐effective, and it produces largely accurate estimates of population allele frequencies (Futschik & Schlötterer, 2010). Thus, statistical methods tailored for E&R studies are especially valuable. Notably so when investigating allele frequency trajectories originating from pooled samples of small populations.

Several statistical approaches have been proposed to analyse these data and detect signatures of selection across the genome. A few such methods consider allele frequency changes between two time points. These simply identify those loci where there is a consistent difference in frequency between time points. One such approach is the widely‐used Cochran–Mantel–Haenszel (CMH) test (Cochran, 1954). Such tests are often preferred since they are very fast, which makes them suitable for genome‐wide datasets. Other approaches allow for more than two time points: for example, Wiberg et al. (2017) used generalized linear models and introduced a quasi‐binomial distribution for the residual error; and Topa et al. (2015) employed Gaussian Process models in a Bayesian framework to test for selection whilst accounting for sampling and sequencing noise. Whilst the latter methods use more sophisticated statistical approaches, they remain descriptive with respect to the underlying evolutionary processes. In contrast, mechanistic approaches explicitly model evolutionary forces, such as genetic drift and selection. Such models have the advantage that they can properly account for drift, which may generate allele frequency changes that can easily be mistaken for selection. Indeed, this is usually the case for E&R experimental populations with low effective population sizes (*N*_*e*_), where genetic drift is the main evolutionary force determining the fate of most alleles.

The Wright‐Fisher (WF) model is the most used mechanistic model for allele frequencies from time series data. There have been numerous studies that rely on approximations of the WF process, for example, its diffusion limit (Bollback et al., 2008), a one‐step process where there is a finite number of allele frequency states (Malaspinas et al., 2012), a spectral representation of the transition density function (Steinrücken et al., 2014), or a delta method to approximate the mean and variance of the process (Lacerda & Seoighe, 2014). More recently, Kojima et al. (2019) developed an expectation‐maximization (EM) algorithm of the WF model diffusion approximation suited for replicated E&R designs. Others have additionally considered the importance of haplotypes arising in a population via mutation (Illingworth & Mustonen, 2012; Nené et al., 2018), or implemented an approximation to the multi‐locus WF process over tens of generations (Terhorst et al., 2015). Amongst these methods, most infer selection parameters in the form of selection coefficients, whilst some can also estimate the population size, allele age, mutation rate and even the dominance coefficient. Such parameters are key for understanding the process of genetic adaptation. Nonetheless, there are only a few approaches that couple parameter estimation with explicitly testing for selection (Feder et al., 2014; Iranmehr et al., 2017; Taus et al., 2017; Terhorst et al., 2015). Whilst these approaches are useful for detecting selected variants and estimating the strength of selection, not all of them are implemented in software packages that can be used genome‐wide for E&R experiments.

Most approaches assume linkage equilibrium, and consequently, each trajectory is analysed independently from the effects of neighbouring sites. In reality, allele frequencies at linked loci co‐vary which can bias the inference around selected sites. Some have tried to measure the impact of linked selection through analysing autocovariances between adjacent sites (Buffalo & Coop, 2019), and others have investigated the correlation between nearby loci to identify selected haplotypes (Franssen et al., 2017) or used a hidden Markov model with states for two linked loci which accounts for genetic recombination (He et al., 2020). Whilst these efforts are a step in the right direction, neither approaches directly estimate selection coefficients nor do they test for selection. These two approaches do not rely on modelling evolutionary processes explicitly.

To provide a review of methods that are available for analysing E&R experiments, Vlachos et al. (2019) have produced a comprehensive benchmarking analysis of such methods. It features a number of approaches but not all of these methods estimate selection coefficients. Based on Vlachos et al.'s (2019) work, three mechanistic methods are thus particularly relevant in an E&R context: Wright‐Fisher Approximate Bayesian Computation (WFABC, Foll et al. (2015)), Composition of Likelihoods for E&R experiments (CLEAR, Iranmehr et al. (2017)) and LLS (Linear Least Squares, Taus et al. (2017)). These methods differ in how they model drift and selection, the inferential approach to estimate selection coefficients, the hypothesis testing strategy, and the extent to which they consider specific experimental conditions (Table 1). WFABC employs an ABC approach that uses summary statistics to compare simulated and real data. It jointly infers the posterior of both *N*_*e*_ and the selection coefficient at some locus in the genome using allele frequency trajectory simulations. Real and simulated summary statistics must agree to a certain predefined scale. This makes WFABC computationally intensive. CLEAR computes maximum‐likelihood estimates of selection parameters using a hidden Markov model tailored for small population sizes. LLS assumes that allele frequencies vary linearly with selection coefficients such that the slope provides the coefficient estimate. Although all three methods have been shown to accurately estimate selection coefficients, they rely heavily on empirical parameter distributions to perform hypothesis testing: (i) WFABC is highly dependent on how accurately the chosen set of summary statistics describes the underlying evolutionary forces determining the observed trajectories; (ii) CLEAR relies on genome‐wide simulations to calculate an empirical likelihood‐ratio statistic to assess significance; and (iii) LLS computes an empirical distribution of *p*‐values simulated under neutrality. One other common thread amongst these tools is that they do not account for linked selection. Be it background selection or hitchhiking, these software estimate selection without looking into how linked loci might affect other sites' trajectories. Additionally, the four software vary substantially in computational effort. Therefore, currently available methods are still limited in their use for genome‐wide hypothesis testing.

**TABLE 1:** Currently available software for estimating selection coefficients in E&R experiments.^a^

	WFABC	CLEAR	LLS	Bait‐ER
Inference approach	Approximate Bayesian computation	Maximum likelihood	Linear least squares^b^	Bayesian
Hypothesis testing	Bayes factors Depends heavily on summary statistics	Likelihood‐ratio tests Empirical *p*‐values based on genome‐wide drift simulations	Empirical simulated *p*‐values based on simulations of allele trajectories	Bayes factors Based on the posterior distribution
Assumptions	WF model	WF model	WF and Moran model Allele frequencies vary linearly with the selection coefficients Weak selection	Time‐continuous Moran model
Accounts for replicates	No	Yes	Yes	Yes
Accounts for sequencing noise	No	Yes	No	Yes
Reference	Foll et al. (2015)	Iranmehr et al. (2017)	Taus et al. (2017)	This study

Abbreviation: WF, Wright‐Fisher.

^a^ The table describes several features of each method namely: (i) the approach used for inferring selection coefficients, (ii) whether it performs hypothesis testing or not, (iii) what sort of assumptions are made about the underlying population genetics model, (iv) its overall computational and inference performance, (v) whether it accounts for multiple replicate populations, and (vi) whether it accounts for sampling variance due to sequencing noise.

^b^ LLS under the assumption of linearity is equivalent to a maximum likelihood approach.

We propose a new Bayesian inference tool – Bait‐ER – to estimate selection coefficients in E&R time series data. It is suitable for large genome‐wide polymorphism datasets and particularly useful for small experimental populations. As our new approach was implemented in a Bayesian framework, it gives posterior distributions of any selection parameters whilst considering sources of experimental uncertainty. Bait‐ER jointly tests for selection and estimates selection contrary to other state‐of‐the‐art methods. It does not rely on empirical or simulation‐based approaches that might be computationally intensive, and it properly accounts for specific shortcomings of E&R experimental design. As it currently stands, Bait‐ER is not modelling the impact of linked selection. However, to test Bait‐ER and other software, we explore individually simulated trajectories, as well as whole chromosome arm simulations with linkage and an analysis of real data. We show that Bait‐ER is faster than other available software, when accounting for hypothesis testing, and still performing accurately in some particularly difficult scenarios.

## 2 MATERIALS AND METHODS

### 2.1 Method outline

E&R experiments produce a remarkable amount of data, namely allele frequencies for thousands to millions of loci. We created a Bayesian framework to infer and test for selection at an individual locus that is based on the Moran model. It estimates the selection coefficient, *σ*, for each allele frequency trajectory, which relies on the assumption that the variant in question is a potential causative locus. The Moran model is especially useful for studies that have overlapping generations, such as insect cage experimental designs (Figure 1). Such cage experiments are easier to maintain in the lab and allow for larger experimental population sizes avoiding potential inbreeding depression and crashing populations (Kawecki et al., 2012). Furthermore, Bait‐ER combines modelling the evolution of an allele that can be under selection whilst accounting for sampling noise to do with pooled sequencing and finite sequencing depth. Our method takes allele count data in the widely used sync format (Kofler et al., 2011) as input. Each locus is described by allele counts per time point and replicate population. The algorithm implemented includes the following key steps:

1. Bait‐ER calculates the virtual allele frequency trajectories accounting for *N*_*e*_ that is provided by the user. This step includes a binomial, or beta‐binomial, sampling process that corrects for pool‐seq‐associated sampling noise.

2. The log posterior density of *σ* is calculated for a given grid of *σ*‐values. This step requires repeatedly assessing the likelihood function (equation 3 in section 2.2).

3. The log posterior values obtained in the previous step are fitted to a gamma surface (details on surface fitting can be found in Figure S2).

4. Bait‐ER returns a set of statistics that describe the posterior distribution of *σ* per locus. In particular, the average *σ* and the log Bayes Factor (BF) are the most important quantities. In this case, BFs test the hypothesis that *σ* is different from 0. Bait‐ER also returns the posterior shape and rate parameter values, *α* and *β*, respectively. These can be used to compute other relevant statistics (e.g., credible intervals, variance).

### 2.2 Model description

Let us assume that there is a biallelic locus with two alleles, *A* and *a*. The evolution of allele A in time is fully characterized by a frequency trajectory in the state space {*nA*, (*N*−*n*)*a*}, where *n* is the total number of individuals that carry allele *A* (in a population of size *N*). Supposing the allele evolves according to the Moran model where a randomly chosen individual reproduces as another is randomly drawn from the population for death, the transition rates for the process are the following

(1) $$\begin{array}{c} P_{n,n-1}=\frac{n\left(N-n\right)}{N}\\ P_{n,n+1}=\frac{n\left(N-n\right)}{N}\left(1+\sigma \right) \end{array},$$

where 1 + *σ* is the fitness of any *A*‐type offspring and *σ* the selection coefficient for allele *A*. If *σ* = 0, that is, *A* is evolving neutrally, then none of the alleles is preferred at reproduction. Let *X*_*t*_ be the number of copies of *A* in a population of *N* individuals and *x*_*t*_ the observed counts of *A* at that time; the probability of a given allele trajectory ***X*** can be defined using the Markov property as

(2) $$p\left(X|\sigma \right)=p\left(X_{0}=x_{0}\right)\prod_{t=1}^{T}p\left(X_{t}=x_{t}|X_{t-1}=x_{t-1},\sigma \right),$$

where *T* is the total number of time points measured in generations at which the trajectory was assayed. The conditional probability on the left‐hand side of the equation has one calculating $X_{t}=e^{{\mathrm{Qd}}_{t}}X_{t-1}$, where *Q* is the rate matrix defined in (1) and *d*_*t*_ the difference in number of generations between time point *t* and *t*−1. The probability of a single allele frequency trajectory can be generalized for *R* replicates by assuming their independence

(3) $$p\left(X|\sigma \right)=\prod_{r=1}^{R}p\left(X_{0}^{r}=x_{0}^{r}\right)\prod_{t=1}^{T}p\left(X_{t}^{r}=x_{t}^{r}|X_{t-1}^{r}=x_{t-1}^{r},\sigma \right).$$

The main caveat for pool‐seq data is the fact that it provides estimates for allele frequencies, not true frequencies. For that reason, we assume that the allele counts are generated by a binomial or beta‐binomial sampling process which depends on the frequency of allele *A* and the total sequencing depth *C* obtained by pool‐seq. We then recalculate the probability of the Moran states given an observed allele count *c*, which becomes the following with binomial sampling

(4) $$p\left(\left\{\mathrm{nA},\left(N-n\right)a\right\}|\left\{c,C\right\}\right)\propto \left(\begin{array}{c} C\\ c \end{array}\right){\left(\frac{n}{N}\right)}^{c}{\left(1-\frac{n}{N}\right)}^{C-c},\;n=0,\ldots ,N.$$

This step is key for it corrects for sampling noise generated during data acquisition, which is particularly relevant for low‐frequency alleles and poorly covered loci.

### 2.3 Inferential framework

We used a Bayesian framework to estimate *σ*. It requires allele counts and coverage for each time point and replicate population {***c***, ***C***} at each position as input. The posterior distribution can be obtained by

(5) $$p\left(\sigma |\left\{c,C\right\}\right)\propto p\left(\sigma \right)p\left(\left\{c,C\right\}|\sigma \right).$$

Our algorithm is defined using a gamma prior on *σ*. The posterior cannot be formally obtained; hence, we define a grid of *σ* values for which we calculate the posterior density. Estimating the posterior distribution $p\left(\sigma |\left\{c,C\right\}\right)$ is a time‐consuming part of our algorithm because the likelihood is computationally costly to compute. To avoid this burden, we fit the posterior to a gamma density

(6) $$\log p\left(\sigma |\left\{c,C\right\}\right)=c+\left(\alpha -1\right)\log\sigma -\beta \sigma ,$$

where *α* and *β* are the shape and rate parameters, respectively, and *c* the normalization constant. The gamma fitting represents a good trade‐off between complexity, since it only requires two parameters, but its density may take many shapes. As one requires the values of *α* and *β* that best fit the gamma density for further analyses, we find the least squares estimates of *α* and *β* (and *c*), such that the error is minimal. The estimation is as follows

(7) $$\begin{array}{c} \begin{array}{l} \hat{\alpha }=\frac{-\left(s_{2}s_{4}+s_{4}^{2}-s_{6}-s_{7}\right)\left(s_{1}^{2}-s_{8}\right)-\left(s_{3}+s_{1}s_{2}+s_{1}s_{4}+s_{5}\right)\left(s_{1}s_{4}-s_{5}\right)}{s_{7}s_{1}^{2}-2s_{4}s_{5}s_{1}+s_{5}^{2}+s_{4}^{2}s_{8}-s_{7}s_{8}}\;\wedge \\ \hat{\beta }=\frac{-s_{3}s_{4}^{2}+s_{2}s_{5}s_{4}+s_{1}s_{6}s_{4}-s_{5}s_{6}-s_{1}s_{2}s_{7}+s_{3}s_{7}}{s_{7}s_{1}^{2}-2s_{4}s_{5}s_{1}+s_{5}^{2}+s_{4}^{2}s_{8}-s_{7}s_{8}}, \end{array} \end{array}$$

where $s_{1}=\sum_{i}x_{i}/N$, $s_{2}=\sum_{i}y_{i}/N$, $s_{3}=\sum_{i}x_{i}y_{i}/N$, $s_{4}=\sum_{i}\log x_{i}/N$, $s_{5}=\sum_{i}x_{i}\log x_{i}/N$, $s_{6}=\sum_{i}y_{i}\log x_{i}/N$, $s_{7}=\sum_{i}\log^{2}x_{i}/N$ and $s_{8}=\sum_{i}x_{i}^{2}/N$. We evaluated the fitting of the gamma density for neutral and selected loci, and observed that a gamma surface with five points describes the log posterior of selected and neutral loci well (Figure S2).

Bait‐ER was implemented with an allele frequency variance filter that is applied before performing the inferential step of our algorithm. This filtering process excludes any trajectories that exhibit no change or whose allele frequency varies very little throughout the experiment from further analyses. We assess the trajectories' frequency increments and exclude loci with frequency variance lower than 0.01. These correspond to cases where trajectories are statistically uninformative since allele frequencies are essentially constant. Trajectories such as these typically have both inflated $\hat{\sigma }$ and BFs. For bookkeeping, these trajectories are included in the output file, despite Bait‐ER not performing the selection inference step on them. This makes Bait‐ER suitable for large genome‐wide datasets without losing any relevant information on trajectories that might be initially flat but may eventually escape drift very quickly.

Bait‐ER is implemented in C++ and freely available for download at https://github.com/mrborges23/Bait‐ER. Here, we provide a tutorial on how to compile and run Bait‐ER, including a toy example with 100 loci taken from Barghi et al. (2019).

### 2.4 Simulated data

We tested our algorithm's performance under several biologically relevant scenarios using (1) a Moran model allele frequency trajectory simulator, and (2) the individual‐based forward simulation software MimicrEE2 (Vlachos & Kofler, 2018).

The Moran model simulator was used to benchmark Bait‐ER's performance across a range of experimental conditions, and to compare our estimates of *σ* to those of CLEAR (Iranmehr et al., 2017), EMWER (Kojima et al., 2019), LLS (Taus et al., 2017) and WFABC (Foll et al., 2015). Experimental designs included those with varying coverage (20×, 60× and 100×), number of replicate populations (2, 5 and 10) and number of sampled time points (2, 5 and 11). In addition to simulating even sampling throughout the experiment, we tested our method on trajectories where we varied sampling towards the start or towards the end of said experiment. Total study length might also affect Bait‐ER's estimation; therefore, we tracked allele frequency trajectories for 0.2*N*_*e*_ and 0.4*N*_*e*_ generations. A full description of these parameters can be found in Table 2.

**TABLE 2:** Simulated scenarios.

Parameter	Simulated values	Notes
Population parameters		
Effective population size (*N*_*e*_)	100, **300** and 1000	Representing a small, a typical and a large in E&R study population
Allele's initial frequency (*p*_0_)	**0.01, 0.05, 0.1 and 0.5**	Representing rare, low‐frequency and common alleles
Selection coefficient (*σ*)	0.1/10*N*_*e*_, 1/*N*_*e*_ and 10/*N*_*e*_	Representing regimes of neutrally evolving, drift‐dominated, and selection‐dominated allele trajectories
Experimental parameters		
Coverage (C)	20×, **60**× and 100×	Low, medium and high coverage for pool‐seq data
Number of replicates (*R*)	2, **5** and 10	
Number of time points (*T*)	2, **5** and 11 time points, assessed at generations	Represents different combinations of total number of time points, experiment lengths and distribution of sampling events (uniform/non‐uniform)
	(0.0, 0.2),	
	**(0.00, 0.05, 0.10, 0.15, 0.20)**,	
	(0.00, 0.04 0.08 0.12 0.20),	
	(0.00, 0.08 0.12 0.16 0.20),	
	(0.0, 0.1 0.2 0.3 0.4) and	
	(0.00, 0.02 0.04 0.06 0.08 0.10 0.12 0.14 0.16 0.18 0.20) relative to *N*_*e*_.	

*Note*: The simulated parameters can be divided into two categories: Those which are related with the population dynamics (effective population size, selection coefficient, and allele's starting frequency) and those related to the experimental design (coverage, number of time points and number of replicates). To test the experimental conditions, we defined a base experiment with 5 replicates, 5 uniformly distributed time points (total span of 0.20*N*_*e*_ generations) and a coverage of 60×. This base experiment is highlighted in bold. The two maximum experiment lengths considered (0.2*N*_*e*_ and 0.4*N*_*e*_) were chosen based on typical E&R experimental designs. Illustrative trajectories of some of the simulated scenarios are represented in Figure S3.

To compare Bait‐ER to other software, we used experimental parameters that resemble realistic E&R designs. Our base experiment populations consist of 300 individuals that were sequenced to 60x coverage. Five replicate populations were evenly sampled five times throughout the experiment. We then simulated 100 allele frequency trajectories for all starting frequencies and selection coefficients mentioned above. We simulated trajectories for 0.25*N*_*e*_ as well as 0.5*N*_*e*_ generations.

The performance of both CLEAR, EMWER, and LLS was assessed by running the software with a fixed population size of 300 individuals (–*N* = 300, pop:300 and estimates(…, *Ne* = 300), respectively). To estimate selection coefficients under the LLS model, we used the estimateSH(…) function assuming allele codominance (h = 0.5). WFABC was tested with a fixed population size of *N*_*e*_ individuals (‐n 300), lower and upper limit on the selection coefficient of −1 and 1, respectively (min_s −1 and ‐max_s 1), maximum number of simulations of 10 000 (‐max_sims 10 000) and four parallel processes (‐n_threads 4). The programme was run for 1200 s, after which the process timed out to prevent it from running indefinitely in case it fails to converge. This caused trajectories with starting allele frequencies of 5% and 1% not to be analysed at all. We have thus only been able to include results for alleles starting at 10% and 50% frequencies. See Table 3 for details on software input format, functions and scripts as well as a list of all parameters used in the analysis.

**TABLE 3:** Software usage for Moran trajectory analysis.

Usage	Bait‐ER	CLEAR	EWMER	LLS	WFABC
Input file format	sync	sync	sync	sync	Programme‐specific format
Function(s) used	Baiter C++ script	CLEAR.py python script	EMWER.py python script	Read in sync file: read. sync()	wfabc_2 C++ script
				Extract allele frequency trajectories: af.traj()	
				Infer selection: estimateSH()	
Parameter(s) used	Parameters in config file	–*N* = 300	Parameters in condition file	h = 0.5	‐fixed_N 300
	Population_size 300		pop:300	*N*_*e*_ = 300	‐min_s −1
	Prior_parameters 0.001, 0.001		slc:0		‐max_s 1
			dom:0.5		‐max_sims 10 000
			opt:s		
			disc:100		
			rbd:0.01, 0.99		
			allele: positive		

*Note*: Input file format, functions and scripts as well as parameters used for analysing Moran model‐generated trajectories with Bait‐ER, CLEAR, EMWER, LLS and WFABC. Parameters are according to base experiment conditions defined in Table 2.

Finally, we used data simulated with MimicrEE2 (Vlachos & Kofler, 2018) by Vlachos et al. (2019) to benchmark Bait‐ER and compare it extensively with other relevant statistical methods. MimicrEE2 is a Wright‐Fisher simulation software that allows for whole chromosomes to be simulated under a wide range of parameters mimicking as well as the effects of linkage on allele frequencies (see also Figures S18–S23, S27 and S28 for a comparison of population parameters, including nucleotide diversity, with real experimental data). This simulated dataset consisted of 10 replicate experimental populations, and each experimental population consisted of 1000 diploid organisms evolving for 60 generations. The haplotypes used were based on 2L chromosome polymorphism patterns from real *Drosophila melanogaster* fly populations (Bastide et al., 2013). Recombination rate variation was based on the *D. melanogaster* recombination landscape (Comeron et al., 2012). Here, 30 segregating loci were randomly picked to be targets of selection. Sites were initially segregated at a frequency between 0.05 and 0.95.

Benchmarking Bait‐ER using the data described above allowed us to look into our method's robustness when the data generating model is not Moran: the first scenario includes allele frequency trajectories simulated under a Wright‐Fisher model of a selective sweep; and the second consists of trajectories simulated under a quantitative trait model with truncating selection. In the former, each of the targets of selection was simulated with a selection coefficient of 0.05. For the latter, 80% of the individuals with the largest trait values were chosen to reproduce.

### 2.5 Application

We applied our algorithm to the published dataset from an E&R experiment in 10 replicates of a *Drosophila simulans* population to a hot temperature regime for 60 generations (Barghi et al., 2019). Populations were kept at a census size of 1000 individuals. The experimental regime consisted of light and temperature varying every 12 h. The temperature was set at either 18°C or 28°C to mimic night and day, respectively. The authors extracted genomic DNA from each replicate population every 10 generations using pool‐seq. The full dataset consists of more than 5 million SNPs. We subsampled the data such that Bait‐ER was tested on 20% of the SNPs. Subsampling was performed randomly across the whole genome.

## 3 RESULTS

### 3.1 Impact of E&R experimental design on detecting targets of selection

**FIGURE 2:**
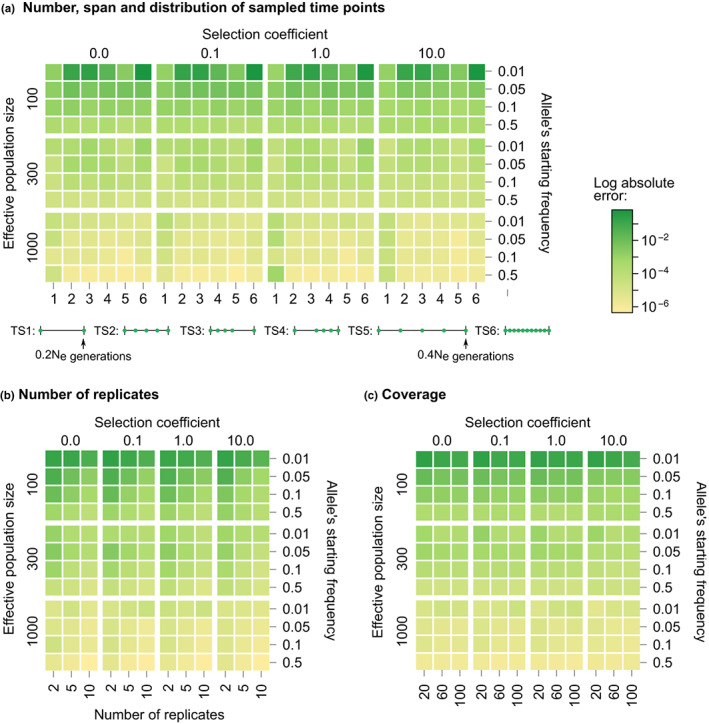
Impact of E&R experimental design on *N*_*e*_‐scaled estimated selection coefficients. Each square of the heatmap represents the error of the scaled estimated selection coefficients, that is, the log of the absolute difference between the estimated and the true simulated $\sigma_{s}:\log\left(\hat{\sigma }-\sigma \right)$, for a range of population dynamics and E&R experimental conditions. (a) Number, span and distribution of sampled time points. The six time schemes differ according to the following criteria: Most time schemes have five sampling events, except for TS1 and TS6, which have two and 11 time points, respectively; all time schemes have a total span of *N*_*e*_/5 generations, except for TS5, which has double the span (2*N*_*e*_/5); uniform sampling was used in most scenarios but for TS3, which is more heavily sampled during the first half of the experiment, and TS4, during the second half. The two maximum experiment lengths considered (0.2*N*_*e*_ and 0.4*N*_*e*_) were chosen based on typical E&R experimental designs. (b) Number of replicates. (c) Coverage. To test the experimental conditions, we defined a base experiment with five replicates, five uniformly distributed time points (total span of 0.20*N*_*e*_ generations) and a coverage of 60×. The complete set of results is shown in Figures S4–S7.

Bait‐ER not only models the evolution of allele frequency trajectories but it also considers aspects of the experimental design specific to E&R studies. Bait‐ER can thus be used to gauge the impact of particular experimental conditions in pinpointing targets of selection. We simulated allele frequency trajectories by considering a range of experimental parameters, including the number and span of sampled time points, the number of replicated populations, and coverage. Each of these settings was tested in different population scenarios that we defined by varying population size, starting allele frequency, and selection coefficient. We assessed the error of the estimated selection coefficients by calculating the absolute bias in relation to the true simulated value. In total, we investigated 576 scenarios (Table 2). Heatmaps in Figure 2a–c show the error for each scenario.

Heatmaps a, b, and c in Figure 2 show that the initial frequency is a determining factor in the accuracy of $\hat{\sigma }$ in E&R experiments. We observed that trajectories starting at very low frequencies (around 0.01) may provide unreliable estimates of *σ*. However, $\hat{\sigma }$'s accuracy can be improved by either increasing the sequencing depth (Figure S6) or the number of replicates (Figure S5). Sequencing depth and replication have also been found to affect other selection inference methods (e.g., Kofler & Schlötterer, 2014 and Taus et al., 2017). Designs with high coverage and several replicates may be appropriate when potential selective loci appear at low frequencies (e.g., dilution experiments). Surprisingly, alternative sampling schemes do not seem to substantially impact the accuracy of *σ* (Appendix S1). These results have practical importance because sampling additional time points is time‐consuming and significantly increases the cost of E&R experiments.

#### 3.1.1 A note on population size

When using Bait‐ER to estimate selection coefficients, one needs to specify the effective population size, *N*_*e*_. However, as effective population size and strength of selection are intertwined, misspecifying *N*_*e*_ will directly affect estimates of selection. *N*_*e*_ is often not known at the start of the experiment, but plenty of methods can estimate it from genomic data, for example, Jónás et al. (2016). To assess the impact of misspecifying *N*_*e*_ on *σ* posterior, we simulated allele frequency trajectories using a fixed population size of 300 individuals. We then ran Bait‐ER setting the effective population size to 100 or 1000. By doing so, we are increasing and decreasing, respectively, the strength of genetic drift relative to the true simulated population.

**FIGURE 3:**
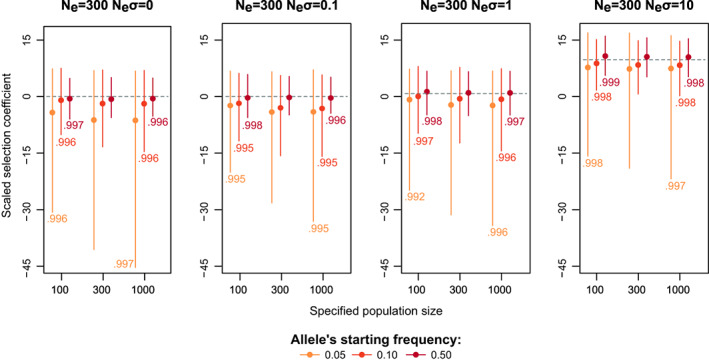
Impact of the user‐specified population size on the estimation of *N*_*e*_‐scaled selection coefficients. The plots show the distribution of the estimated *N*_*e*_‐scaled selection coefficients where the population size is misspecified. Vertical lines and points indicate the interquartile range and median scaled selection coefficient. Each plot represents a specific scenario that was simulated by varying the population size, the true scaled simulated selection coefficient (indicated within brackets (*N*_*e*_, *N*_*e*_σ)) and starting allele frequency (indicated by the yellow‐to‐red colour gradient). The numbers next to each bar correspond to the Spearman's correlation coefficient, which correlates the BFs of the 100 replicated trajectories between the cases where we have either under‐ and overspecified the population size (*N*_*e*_ = 100 or 1000, respectively) and the case where we use the true population size (*N*_*e*_ = 300). Regarding simulated experimental design, we defined a base experiment with five replicates, five uniformly distributed time points (total span of 0.20 *N*_*e*_ generations) and a coverage of 60×.

Bait‐ER produces highly accurate estimates of *σ* regardless of varying *N*_*e*_ (Figure 3 and Figure S7). Misspecifying it merely rescales time in terms of Moran events rather than changing the relationship between *N*_*e*_ and the number of Moran events in the process. Further, we observed that the BFs are generally higher when the specified *N*_*e*_ is greater than the true value, suggesting an increased false positive rate. The opposite pattern is observed when the population size one specifies is lower than the real parameter. Additionally, we investigated the relationship between BFs computed with the true *N*_*e*_ and those produced under a misspecified *N*_*e*_. We found that these BFs are highly correlated (Spearman's correlation coefficients were always higher than 0.99; Figure 3 and Figure S7). Taken together, our results indicate one should use a more stringent BF acceptance threshold if estimates of the experimental *N*_*e*_ have wide confidence intervals.

Furthermore, we assessed Bait‐ER's computational performance by comparing the relative CPU time whilst varying several user‐defined experimental parameters. We found that increasing *N*_*e*_ affects our software's computational performance most substantially (31‐fold increase in CPU time when increasing the simulated population size from 300 to 1000 individuals; Table S1).

### 3.2 Benchmarking Bait‐ER with LLS, CLEAR and WFABC

#### 3.2.1 Simulated Moran trajectories

**FIGURE 4:**
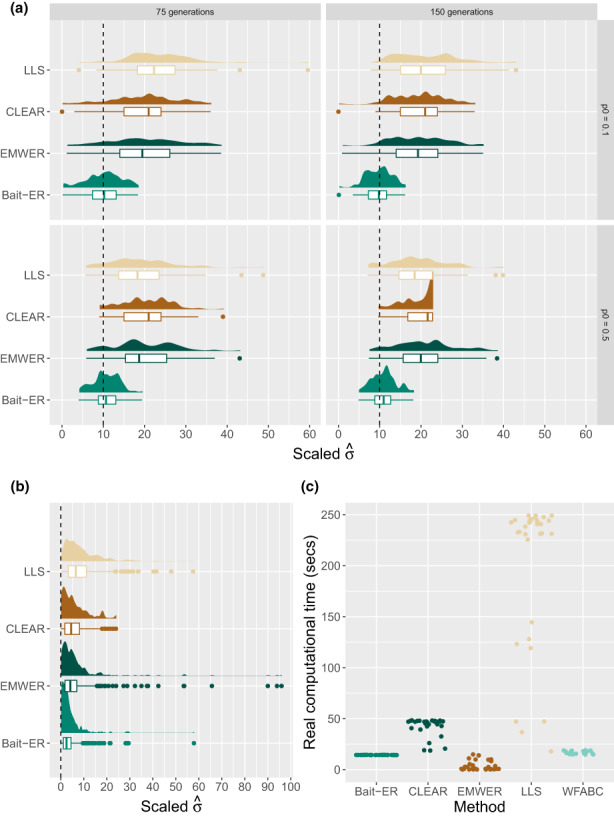
Comparison of selection inference performance on individual Moran allele frequency trajectories. (a, b) *N*_*e*_‐scaled *σ* estimates produced by Bait‐ER, CLEAR, EMWER and LLS. Results are shown for trajectories with starting frequencies of 10% and 50%. (a) Strongly selected alleles (*N*_*e*_ *σ* = 10) with separate panels for 75 (left) and 150 generation long trajectories and 10% (top) and 50% (bottom) starting frequencies. (b) Only neutrally evolving (*N*_*e*_ *σ* = 0) alleles were considered here. These are combined distributions for the same two simulation lengths (75 and 150 generations) as well as the two starting frequencies. LLS returned NA's for 3 out of 800 trajectories which were excluded from these graphs. (c) Real computational time for Bait‐ER and the other four approaches tested. From left to right, computational time in seconds including both inference and hypothesis testing for Bait‐ER, CLEAR, EMWER, LLS, and WFABC is shown here.

To compare the performance of Bait‐ER to that of other relevant software, we set out to simulate Moran frequency trajectories under the base experiment conditions described above. We tested Bait‐ER as well as CLEAR (Iranmehr et al., 2017), EMWER (Kojima et al., 2019), LLS (Taus et al., 2017) and WFABC (Foll et al., 2015) on 100 trajectories for four starting frequencies (from 1% to 50%) and four scaled selection coefficients (0 ≤ *N*_*e*_ ≤ 10). All population parameters were tested for both 75 and 150 generations of experimental evolution. Figure 4a,b show the *σ* estimates for Bait‐ER, CLEAR under two starting frequency scenarios – 10% and 50% – and two *N*_*e*_ *σ* – 0 and 10. Under strong selection (*N*_*e*_ *σ* = 10), CLEAR, EMWER and LLS medians largely agree with each other (see Figure 4a). However, these are upwardly biased in comparison to Bait‐ER's estimates, which are closer to the true value. Under neutrality (Figure 4b), the four methods largely agree with Bait‐ER showing higher accuracy. Overall, Bait‐ER is the method with the smallest variation regardless of the starting frequency and true selection coefficient. On the other hand, WFABC systematically disagrees with Bait‐ER's estimates because its distribution is very skewed towards high *N*_*e*_ *σ* (>180; see Figure S8). This is perhaps unsurprising given that WFABC does not consider replicate populations nor finite sequencing depth unlike the other three methods. We have included WFABC in our study to compare Bait‐ER with another Bayesian method. However, WFABC was not designed for E&R experiments with multiple replicates, hence its poor performance.

Regarding computational performance, Bait‐ER seems to be amongst the fastest methods, even though it is comparable to WFABC and surpassed by EMWER at higher starting frequencies. Bait‐ER's performance is comparable (see Figure 4b), but we tested it on the first replicate population data rather than the five experimental replicates used for the remaining methods. Additionally, WFABC does not provide any statistical testing output such as a Bayes Factor. In contrast, CLEAR and LLS are slower than the other three approaches. Whilst CLEAR takes less than 40 s on average to analyse 100 sites, LLS is the slowest of the four, averaging around 4 min. Overall, these results suggest Bait‐ER is just as accurate and potentially faster than other currently available approaches, which makes it a good resource for testing and inferring selection from genome‐wide polymorphism datasets.

#### 3.2.2 Complex simulation scenarios with recombination

For a more comprehensive study of Bait‐ER's performance, we have analysed a complex simulated dataset produced by Vlachos et al. (2019). The authors simulated an E&R experiment inspired by the experimental set‐up of Barghi et al. (2019) and used polymorphism data from a *Drosophila melanogaster* population. Vlachos et al. (2019) have produced this dataset to standardize software benchmarking by simulating a series of experimental scenarios that are relevant in an E&R context. We have used it to assess Bait‐ER's performance at inferring selection under linkage and varying recombination rates. In particular, we choose to focus on the classic sweep scenario as well a quantitative trait model with truncating selection, which are two of three complex scenarios simulated in Vlachos et al. (2019). Each experiment was replicated 100 times and had 30 targets of selection randomly distributed along a 16Mbp‐long chromosome arm.

**FIGURE 5:**
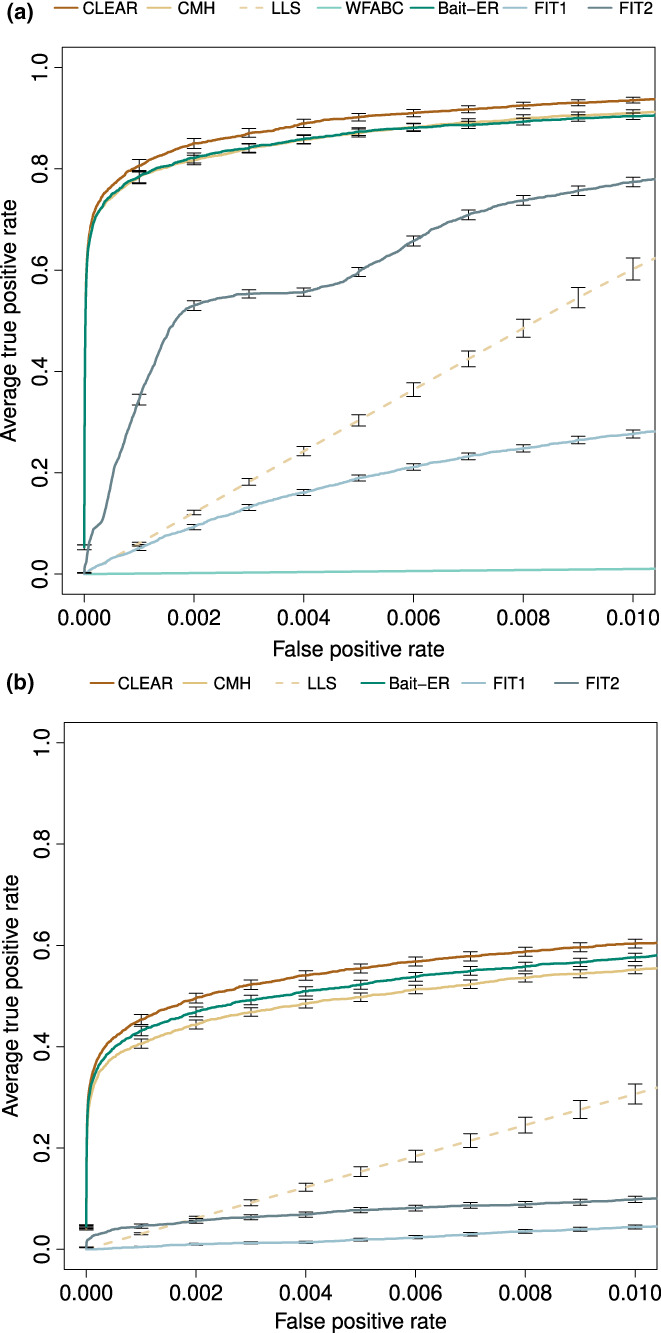
Performance of Bait‐ER and other software at testing for selection in data simulated by Vlachos et al. (2019). ROC (receiver operating characteristic) curves for Bait‐ER, CLEAR, CMH, LLS, WFABC, FIT1 and FIT2 under (a) the classic sweep scenario and (b) a scenario with truncating selection. Note that LLS and WFABC were run on a subset of SNPs in (a), and that WFABC was not included in (b) for it was prohibitively slow and only finished runs for 29 replicate experiments.

ROC (Receiver Operating Characteristic) curves are compared for seven methods, Bait‐ER, CLEAR, the CMH test (Agresti, 2003), LLS and WFABC, as well as FIT1 and FIT2 (Feder et al., 2014), similar to Figure 2a in Vlachos et al. (2019). FIT1 and FIT2 both use a t‐test for allele frequencies and are inaccurate in a classical sweep dataset. Bait‐ER performs well with an average true positive rate of 80% at a 0.2% false positive rate (Figure 5a). Its performance is as good as the CMH test's, but it underperforms slightly in comparison to CLEAR. Bait‐ER, CLEAR and the CMH test greatly outperform LLS and WFABC. We note that Bait‐ER's inferential framework assumes that each biallelic locus is codominant. This is an alternative to estimating dominance parameters which requires a diploid model. CLEAR, EMWER, LLS and WFABC allow for estimating dominance. However, a robust estimate is largely dependent on the dataset. A similar picture to that of the sweep simulation emerges for the truncating selection scenario (Figure 5b). Bait‐ER is amongst the top three methods despite the generating quantitative trait model being misspecified during inference; it is only slightly outperformed by CLEAR.

To assess why Bait‐ER seems to be outperformed by CLEAR, we further investigated CLEAR's selection coefficient estimates. We note that Bait‐ER assumes a continuous‐time Moran model, whilst CLEAR uses a WF model for inference, much like the simulated data analysed here. Comparison of selection coefficients estimated by Bait‐ER and CLEAR showed that Bait‐ER is slightly more accurate on average at estimating true targets' *σ* (Figure S9). In addition, those trajectories that scored highest with CLEAR also produced the highest Bait‐ER *σ* (Figure S10). True targets of selection mostly score in the top half of Bait‐ER's *N*_*e*_ *σ* scale (Figure S25). Overall, Bait‐ER and CLEAR perform to a similar high standard. However, the frequency variance filter implemented in Bait‐ER seems to explain our method's slight underperformance shown in Figure 5. Despite having excluded fewer than 70 (out of 300) targets of selection, Bait‐ER's filtering step has also classified approximately the same amount of neutral trajectories for being too flat for inferring selection. Whilst the two method's false‐positive rates seem to be comparable, Bait‐ER excluded a few selected sites from further analyses as they had changed very little in frequency throughout the experiment.

### 3.3 Analysing E&R data from hot adapted *Drosophila simulans* populations

We have applied Bait‐ER to a real E&R dataset that was published by Barghi et al. (2019). The authors exposed 10 experimental replicates of a *Drosophila simulans* population to a new temperature regime for 60 generations. Each replicate was surveyed using pool‐seq every 10 generations. This dataset is particularly suited to demonstrate the relevance of our method as Barghi et al. (2019) observed a strikingly heterogeneous response across the 10 replicates. The highly polygenic basis of adaptation has proved challenging to measure and summarize thus far.

**FIGURE 6:**
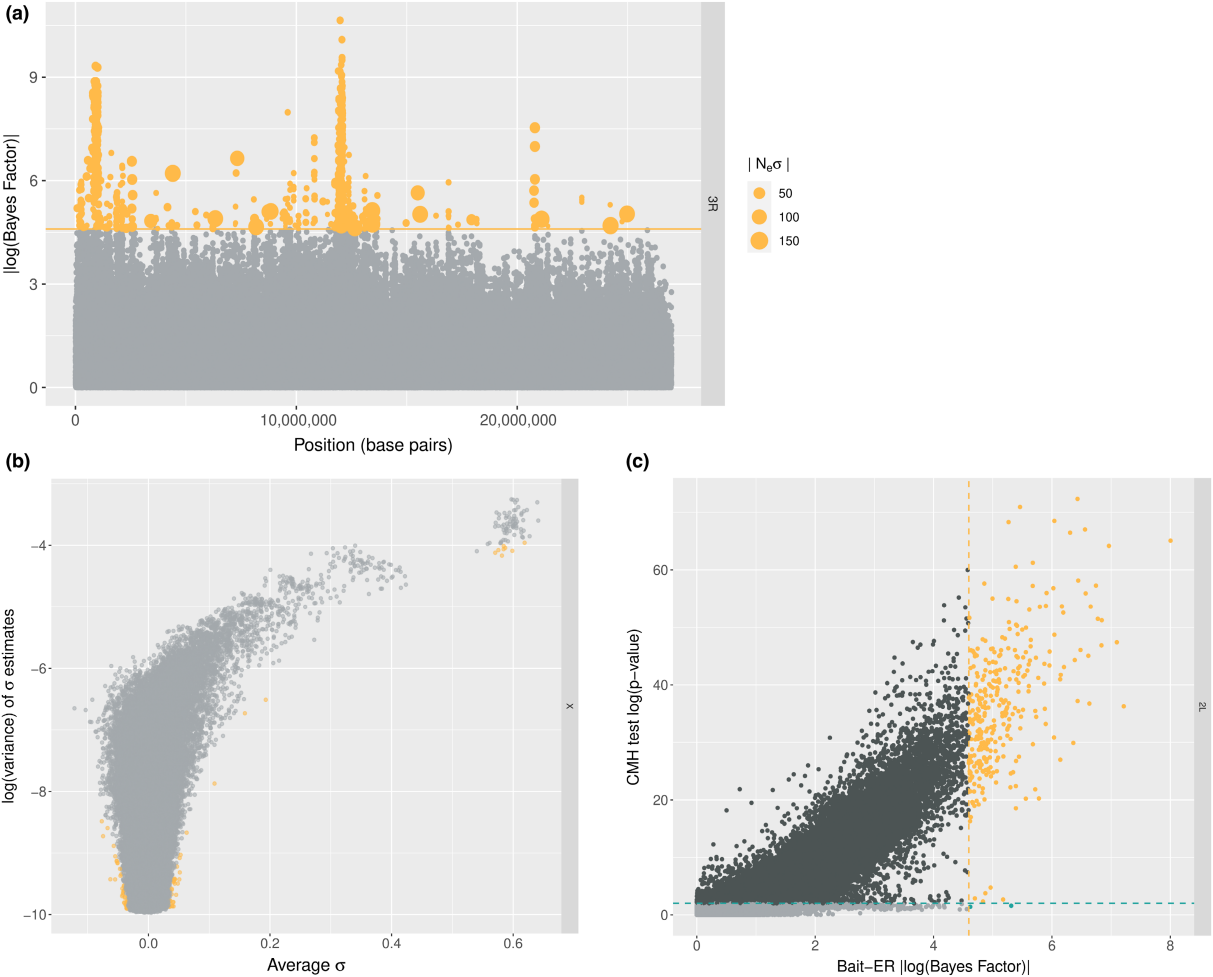
Analysis of Barghi et al. (2019) allele frequency trajectories. (a) Bayes factors on chromosome 3R. The orange line indicates a conservative threshold of 4.6, an approximation which corresponds to log(0.99/0.01), meaning all points in orange have very strong evidence for these to be under selection. The SNPs that are significant at this level are sorted by size according to how strong Bait‐ER's selection coefficients are, that is, points are sized according to how strong the selection coefficient is estimated to be. (b) Variance versus mean sigma on the X chromosome. This graph compares log transformed variances in *σ* estimates to average *σ*s. the variance is calculated using the inferred rate and shape parameters for the beta distribution, and the average *σ* is the mean value of the posterior distribution estimated by Bait‐ER. Orange‐coloured points are significant at a conservative BF threshold of 4.6. (c) Bait‐ER's Bayes factors versus CMH test's *p*‐values on chromosome 2L. Orange‐coloured points correspond to BFs which are greater than 4.6 and *p*‐values ≤ 0.01, that is, those that are considered significant by both tests. Blue coloured points indicated that the computed BF is greater than our threshold, but not significant according to the CMH test. Dark grey points are significant according to the CMH test, but not to Bait‐ER, and light grey points are inferred not significant by both tests.

The *D. simulans* genome dataset is composed of six genomic elements: chromosomes 2–4 and chromosome X. For each element, we have estimated selection parameters using Bait‐ER (mean $\hat{\sigma }$ distributions can be found in Figure S11). Figure 6a shows a Manhattan plot of BFs for the right arm of chromosome 3. We can observe that there are two distinct peaks across the chromosome arm that seem highly significant (BF > 9). These two peaks – one at the start and another just before the centre of the chromosome – should correspond to regions harbouring loci that responded strongly to selection in the new lab environment. Such regions display a consistent increase in frequency across replicate populations (see Figure S24 for the relationship between allele frequency changes and *σ*). Overall, there are only a few other regions that exhibit very strong evidence for selection across the genome (Figure S12). Those are located on chromosomes 2L, 2R and 3L. When compared to the CMH test results as per Barghi et al., Bait‐ER's most prominent peaks seem to largely agree with those produced by the CMH (see Figure S13). The same is true for high BF regions on chromosomes 2L and 2R where there are similarly located *p*‐value chimneys at the start of these genomic elements (Figure S14). Both Bait‐ER and the CMH test did not produce clear signals of selection on chromosomes 3L, 4 and on the X.

One of the advantages of Bait‐ER is that we have implemented a Bayesian approach for estimating selection parameters, which means we can calculate both the mean and variance of the posterior distributions. To examine both of these statistics, we looked into how the posterior variance varies as a function of mean *σ*. Figure 6b shows the relationship between variance and mean selection coefficient for the X chromosome. We observe that the highest mean values also correspond to those with the highest variance. This suggests that the strongest response to selection, that is, the highest estimated *σ* values, is also those showing a fairly heterogeneous response across replicates. The remaining genomic elements seem to show similar patterns, apart from chromosome 4 (see Figure S15). This is consistent with other reports that inferring selection on this chromosome is rather difficult due to its size and low levels of polymorphism (Jensen et al., 2002).

Finally, we compared the *p*‐values obtained by Barghi et al. (2019) and the BFs computed by Bait‐ER. Barghi et al. (2019) performed genome‐wide testing for targets of selection between first and last time points using the CMH test. The tests seem to largely agree for the most significant BFs correspond to the most significant *p*‐values. However, Bait‐ER appears to be more conservative than the CMH test. This follows from the finding that there is quite a substantial proportion of loci (<10% of all loci) that are significant at a *p*‐value threshold of 0.01, which are not accepted as such by Bait‐ER (e.g., chromosome 2L in Figure 6c). Similar patterns are found in other chromosomes (Figure S16).

## 4 DISCUSSION

One of the main aims of E&R studies is to find targets of selection in genome‐wide datasets. For that, we developed an approach that uses time series allele frequency data to test for selection. It does so whilst estimating selection parameters for individual loci. As Bait‐ER does not rely on simulations for statistical testing, it sets itself apart from other currently available methods. Bait‐ER's implementation of the time‐continuous Moran model makes it especially suitable for experimental set‐ups with overlapping generations. In addition, we designed Bait‐ER to be well suited for small population experiments where genetic drift has a substantial impact on the fate of polymorphisms. This is because random frequency fluctuations can force alleles to be more readily lost and, thus, overlooked by selection. When considering such polymorphisms, our stochastic modelling approach to describing their frequency trajectory is most fitting. We assume that the effect of drift is pervasive and that there is added noise from sampling a pool of individuals from the original population. We show that Bait‐ER is faster and just as accurate as other relevant software. Overall, these features make it a desirable approach that can be used in many E&R designs.

We carried out a comprehensive analysis of simulated trajectories where we explored the parameter space for coverage, number of experimental replicates, user‐defined population size, starting allele frequency and sampling scheme (Figure 2; Figure S7). Our results suggest that Bait‐ER's inference is mostly affected by low starting allele frequencies. This can be overcome should the sequencing depth or the number of experimental replicates be increased. Our simulations show that Bait‐ER estimates selection coefficients accurately even if an allele's starting frequency is low but provided coverage is high and there are at least 5 replicates (Figure 2). Although increasing the number of replicates increases the cost of setting up an E&R experiment substantially, improving sequencing depth is certainly within reach. This interesting result might help guide future research. Encouragingly, Bait‐ER performed well with small manageable population sizes, suggesting replication is key, but large populations are not necessarily required for achieving good results.

We also assessed Bait‐ER's performance on a complex chromosome arm dataset simulated by Vlachos et al. (2019). We then compared it to other selection inference programmes of which most are suited for time series allele frequency data. Despite numerous similarities, they vary substantially in terms of model assumptions and what sort of experimental set‐up they are a good fit for. For example, WFABC seems to underperform in comparison with the other methods for E&R experiments. This is likely to be the case because it was modelled for relatively large populations. As Foll et al. (2015) show in their original study, WFABC is accurate for population sizes of 1000 individuals and for both weak and strong selection coefficients. Despite this being low in comparison to experiments in bacteria or yeast, which easily range from 10^5^ to 10^8^, that is not the standard population size we consider in our work. Bait‐ER has been shown to perform well for such large populations (see bottom rows of each graph in Figure 2), as well as small census sizes. In reality, *N*_*e*_ is predicted to be a lot smaller than the census sizes reported in typical E&R studies. Similar to Bait‐ER, CLEAR and LLS are better suited for small population analyses. Whilst CLEAR accounts for uneven coverage, LLS only considers consistency between experimental replicates. In terms of overall performance, Bait‐ER and CLEAR are similar in accuracy but Bait‐ER runs substantially faster. This indicates that inferring selection from WF trajectories simulated with MimicrEE2 produces similar results regardless of whether a WF or a Moran model is used to describe the evolution of such trajectories.

We used ROC curves to compare Bait‐ER's performance to six other methods'. They serve the purpose of showing the performance of a binary classification model at all significance thresholds, regardless of the statistical measurement used, may it be a *p*‐value or a BF. ROC curves address whether the method places the true targets of selection amongst its highest scoring hits. Whilst this is informative, it fails to account for the importance of finding an adequate significance threshold when analysing experimental data. For example, Figure 5 suggests that Bait‐ER and the CHM test perform very similarly. However, the CMH test returns more potential targets than Bait‐ER when comparable thresholds are used for both methods (e.g., Figure 6c that shows the comparison between Bait‐ER logBFs and CMH test *p*‐values for a real *D. simulans* dataset). This is consistent with other reports of the CMH test producing overinflated false positive rates on account of it confounding heterogeneity across replicates with a main effect (Wiberg et al., 2017). Additionally, whilst the CMH might be more prone to identifying high coverage sites, Bait‐ER is not affected by sequencing depth (Figure S26). Altogether, this indicates that Bait‐ER is more conservative and that the CMH test is more prone to producing false positives.

To investigate Bait‐ER's ability to detect selected sites in a real time series dataset, we analysed the *D. simulans* E&R experiment by Barghi et al. (2019). Bait‐ER performs well on this dataset as it is rather conservative and produces only a few very significant peaks across the genome, which suggests it has a low false positive rate. It was designed to account for strong genetic drift, hence the use of a discrete‐population state space. Most of the genome produced BFs greater than 2, indicating that there is not enough resolution to narrow down candidate regions to specific genes despite those very significant peaks. Barghi et al. (2019) argue that there is strong genetic redundancy caused by a highly polygenic response to selection in their experiment. Despite Bait‐ER modelling sweep‐like scenarios rather than the evolution of a quantitative trait using an infinitesimal model, the somewhat elevated BF signal across the genome might indicate that the genetic basis of adaptation to this new temperature regime is indeed polygenic. Our results also suggest that the impact of linked selection might be non‐negligible and worth investigating further.

Linkage between selected and neutral variants has long been shown to cause skewed neutral site frequency spectra (Fay & Wu, 2000). Our analysis of the Barghi et al. (2019) experiment indicates that linked selection might be the cause of a similar skew in this dataset. Of the six genomic elements in the *D. simulans* genome, five show significant SNPs all throughout the chromosome. In an independent study, Buffalo and Coop (2020) have analysed temporal covariances in Barghi et al.'s dataset to quantify the impact of linked selection in a model of polygenic adaptation. They found that the covariances between adjacent time points are positive but do decay towards zero as one examines more distant time intervals. This would be predicted if directional selection is determining the fate of linked neutral loci. Over 20% of genome‐wide allele frequency changes were estimated to be caused by selection, particularly linked selection. Linked selection is likely to have a substantial impact on genome scans such as Bait‐ER that assume independence between sites. This is especially evident in highly significant peaks of BFs (Figure 6; Figure S12). The trajectories within such peaks will have similar sweep‐like shapes and will likely consist of causative loci as well as closely linked neutral sites. These results are in contrast to what we obtained from analysing Vlachos et al. (2019) where linked selection does not generally affect Bait‐ER's ability to detect the true targets of selection (Figure S17). This indicates that the data simulated by Vlachos et al. (2019) might not fully reproduce the complexity of real genomic data.

Barghi et al. (2019) claim that their experiment showed a very distinctive pattern of heterogeneity amongst replicate populations. Buffalo and Coop (2020) also found that there is a substantial proportion of the initial allele frequency change in response to selection that is shared between replicates in the Barghi et al. (2019) dataset, but this pattern is overturned rapidly. This can be caused by the population swiftly reaching the new phenotypic optimum, thereby hitchhiker alleles spread through the population along with adaptive sites, which reach high frequencies very quickly. These linked loci eventually recombine on to other genetic backgrounds causing linkage to dissipate. The consequences of replicate heterogeneity on genome scans are twofold. On the one hand, different segregating haplotypes could be selected for in different replicates. This will cause genome scans not to find any convergent genotype frequencies. The process is difficult to characterize unless there is sufficient data on the founder haplotypes. Numerous studies have time series data that does not include full sequences of those starting haplotypes, for example, Barghi et al. (2019) and Burke et al. (2014). On the other hand, it is possible that multiple interacting beneficial mutations are already present in the standing genetic variation. Interference between linked selected sites through epistasis can reduce the effectiveness of selection (Hill & Robertson, 1966). This will be more prevalent if there are large effect loci in the vicinity. Our results indicate that that might be the case in the sweep simulated by Vlachos et al. (2019), where the authors simulate a little over 10% of the *D. melanogaster* total genome length with 30 selected targets. For moderate to strong selection, that might be enough for linkage to hinder rapid adaptation and produce signatures that are not readily captured in genome scans.

Bait‐ER estimates and tests for selection. However, *σ* estimates are not to be taken literally as linked selection might be inflating individual selection coefficients. Such is the case that nearby sites are not independent from one another that extended haplotypes might be rising to fixation at once. In a short timescale such as that of an evolution study, recombination is unlikely to have had the chance to have broken up haplotypes present in the standing variation. In addition, one expects drift to exacerbate the effect of linked selection in experiments where populations are small. Selection inference methods will likely be affected when the combined effect of linkage and drift is pervasive. Maximum likelihood estimates of selection coefficients were shown to be unaffected by demography in populations as small as 500 individuals (Jewett et al., 2016). However, it is common that *N*_*e*_ in laboratory experiments is lower than the census population size. For example, Barghi et al. (2019) have reared flies in populations of roughly 1000 individuals, but they have estimated *N*_*e*_ to be around 300. Collectively, our results suggest that drift should not be neglected as it might inflate selection coefficient estimates since it exacerbates the extent of linked selection. Its impact can be substantial especially for populations with low polymorphism levels.

Regardless of demographic factors, adaptation of complex traits is a challenging process to characterize. This is because trait variation is influenced by numerous genes and gene networks. There is now some evidence in the literature suggesting that polygenic adaptation is key in a handful of laboratory evolution studies (reviewed by Barghi et al., 2020). The genomic signature left by such a complex process is still hard to describe in its entirety even in a replicated experimental design. It depends on numerous factors, including the total number of causative loci and the levels of standing genetic variation. The more polygenic a trait is the more likely linkage between selected sites is to generate extended selected haplotypes. Nevertheless, directional selection will cause some proportion of selected sites to behave as sweep‐like trajectories. It is those that Bait‐ER is aiming to characterize. In short‐term evolution experiments, theoretical studies have shown that a shift in the phenotypic optimum can result in sweep signatures provided the effect size is large (Jain & Stephan, 2017).

Results from genome scans in E&R studies of small populations generally tend to underperform. Since drift is pervasive and LD is extensive, genome scans might suffer from low power and high false‐positive rates. For that reason, we plan to extend Bait‐ER to explicitly account for linkage, which decays with distance from any given locus under selection. Accounting for linkage should help disentangle the effects of local directional selection on specific variants versus polygenic selection on complex traits. Modelling the evolution of linked sites by including information on the recombination landscape will further clarify the contribution of each type of selection.

## AUTHOR CONTRIBUTIONS

CB, RB and CK conceived the idea. RB developed the inferential framework and wrote the software. CB and RB tested the method on simulated data. CB performed the analyses on the Vlachos et al. (2019) and the Barghi et al. (2019) datasets. CB led the manuscript writing and submission steps. All authors contributed to manuscript revision, read and approved the submitted version.

## CONFLICT OF INTEREST

All authors declare that they have no conflicts of interest.

### PEER REVIEW

The peer review history for this article is available at https://publons.com/publon/10.1111/jeb.14134.

## Supporting information


Appendix S1

## Data Availability

No new sequencing data were generated or analysed in support of this research. Bait‐ER has been released as an open‐source programme that can be downloaded from https://doi.org/10.5281/zenodo.7351736. The simulated dataset published by (Vlachos et al., 2019) is available at https://sourceforge.net/projects/erbenchmark/. The *Drosophila simulans* E&R dataset analysed in Real data section (published in Barghi et al., 2019) can be downloaded from https://doi.org/10.5061/dryad.rr137kn.
